# Daylight space debris laser ranging

**DOI:** 10.1038/s41467-020-17332-z

**Published:** 2020-08-04

**Authors:** Michael A. Steindorfer, Georg Kirchner, Franz Koidl, Peiyuan Wang, Beatriz Jilete, Tim Flohrer

**Affiliations:** 1 Space Research Institute, Austrian Academy of Sciences, Lustbühelstraße 46, Graz, A-8042 Austria; 2 GMV at European Space Agency, Camino Bajo de Castillo S/N, Villanueva de la Cañada, 28692 Spain; 3 European Space Agency, Robert-Bosch-Str. 5, Darmstadt, DE-64293 Germany

**Keywords:** Space physics, Space physics, Space physics

## Abstract

Satellite laser ranging allows to measure distances to satellites equipped with retroreflectors in orbits up to 36000 km. Utilizing a higher powered laser, space debris laser ranging detects diffuse reflections from defunct satellites or rocket bodies up to a distance of 3000 km. So far space debris laser ranging was only possible within a few hours around twilight while it is dark at the satellite laser ranging station and space debris is illuminated by the sun. Here we present space debris laser ranging results during daylight. Space debris objects are visualized against the blue sky background and biases corrected in real-time. The results are a starting point for all space debris laser ranging stations to drastically increase their output in the near future. A network of a few stations worldwide will be able to improve orbital predictions significantly as necessary for removal missions, conjunction warnings, avoidance maneuvers or attitude determination.

## Abstract (web summary)

Space debris laser ranging is a technique to measure distances to defunct satellites or rocket bodies in orbits around Earth which was only possible within a few hours around twilight. Here, the authors show the first space debris laser ranging results during daylight while correcting inaccurate predictions using a real-time target detection software.

## Introduction

In 1964 the first satellite (Beacon-B) equipped with a retroreflector was launched^1^. One year later, the first successful satellite laser ranging (SLR) results were achieved having a precision in the order of meters^2^. Since then the SLR technique rapidly advanced strongly connected to the reduction of the laser pulse width from nanoseconds to picoseconds. Simultaneously the detector technology improved; currently microchannel plates and single photon avalanche diodes (SPAD)^3,4^ are used, some SLR stations are also testing nanowire detectors^5^. Nowadays a single shot precision of a few millimeters^6^, up to navigation^7^ and geostationary orbits, is state of the art. A few stations are specializing on lunar laser ranging^8^ or fiber-based transmission optics^9^. Another important development was the usage of lasers with kHz repetition rate^10^. This allows the identification of individual retroreflectors within the data and the determination of spin and attitude parameters of defunct^11,12^ and active^13–15^ satellites. Since the beginning of the new century laser ranging measurements to space debris objects were performed^16^. Lasers with higher pulse energies and nanosecond pulse width were used to allow the detection of diffusely reflected photons measuring the distance with a single shot precision of approximately 1 m^17,18^. These reflected photons are distributed over a large area and can be detected by multiple stations across Europe. During such bi- or multi-static space debris laser ranging measurements one (active) SLR station fires the laser at a space debris target, while one or more (passive) SLR stations can detect the reflected photons, even without the need of operating a laser themselves^19^. Multi-static experiments can improve orbit predictions of space debris objects by up to an order of magnitude^20^. Data fusion with optical measurements could further improve the orbit determination quality^21^. Up to now only a few stations worldwide contribute to space debris measurements, and these are limited to a few hours around sunrise or sunset. Dedicated campaigns which increase the prediction accuracy of space debris objects are hence difficult to implement. The satellite needs to be illuminated by sunlight while it has to be dark at the station. The visual image of the satellite is used to center the target in the field of view of the SLR telescope before starting the SLR search routine. This is necessary to correct two line element^22^ (TLE) space debris predictions which depending on the orbit can have inaccuracies up to 1 km^23^.

Here, we show successful daylight space debris laser ranging. The visualization of the reflected sunlight of space debris during daylight and the simultaneous real-time correction of offsets to the orbit predictions is demonstrated. The presented results should encourage the laser ranging community to participate—with increased performance—in a network of space debris laser ranging stations worldwide being able to rapidly improve orbit predictions of selected targets.

## Results

### Daylight space debris laser ranging procedure

The daylight space debris laser ranging routine consists of the following steps: the tracking of the target is usually started at elevations above 15°. As soon as the target is visible with the 20 cm piggyback telescope the offsets to the predicted path and the time bias are calculated by the real-time detection software and the orbit used for tracking is immediately corrected to center the target within the field of view of the SLR telescope. Additional across-track offsets are corrected by applying pointing offsets to the receive telescope. Varying biases are continuously corrected during tracking, primarily by correcting the time bias. Range biases of the target due to TLE errors can not be estimated via image analysis and the only chance to apply corrections to the predictions is by shifting the activation time of the detector. The closer the activation of the detector is to the arrival time of a reflected photon the higher the chances of detection are. The space debris laser ranging search routine is an iteration process consisting of applying time biases, optically centering the target and experimentally shifting the detector activation times.

### Daylight space debris laser ranging results

**Fig. 1:**
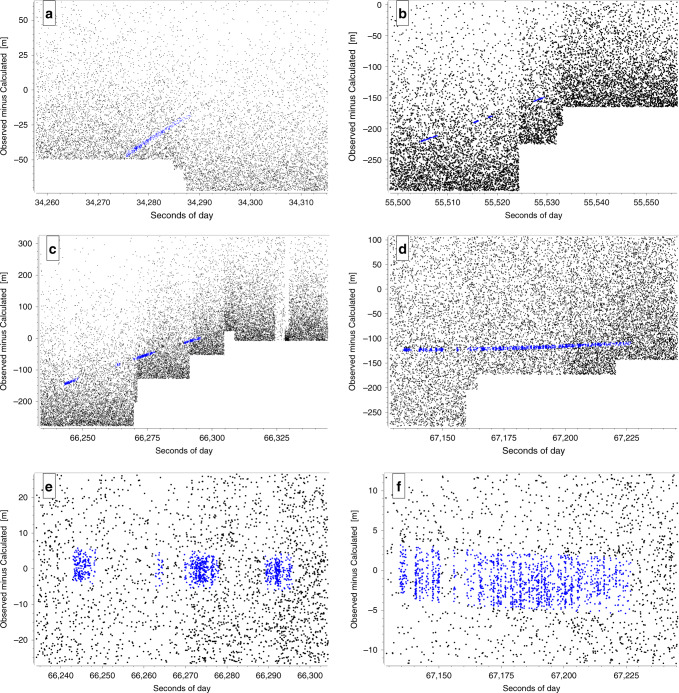
**Daylight space debris laser ranging results**. Observed-minus-calculated residuals [m] of four successful daylight space debris laser ranging passes to rocket bodies at different sun elevations (el_sun_). For **a**–**d** time biases (tb) are applied during post-processing as set during the observations. For **e**, **f**, time biases (tb) and range biases (rb) are corrected during post-processing to match the measurements to the orbit predictions. Identified returns during post-processing are highlighted in blue color. **a** SL-16 R/B (#22285), tb = 9 ms, 2019/03/22, 10:31 (el_sun_ = 39.2°), **b** SL-14 R/B (#20511), tb = −76 ms, 2019/07/24, 20:24 (el_sun_ = 2.0°), **c** SL-16 R/B (#23705), tb = 140 ms, 2019/07/24, 20:38 (el_sun_ = 0.2°), **d** SL-16 R/B (#22803), tb = −150 ms, 2019/10/01, 17:25 (el_sun_ = 11.5°), **e** close up of (**c**), tb = 200 ms, rb = 415 m, **f** close up of (**d**), tb = −158 ms, rb = 133 m.

Four successful space debris passes are presented, which were measured between March and October 2019. Space debris laser ranging measurements were regarded as daylight passes if the elevation of the sun was above the horizon. Three different types of SL (Sea Launch) rocket bodies originating from Zenit, Tsyklon or Vostok launches between 1971 and 1995 were observed^24^. The maximum sun elevation during the measurement was 39° at 10:31 local time on 2019/03/22. The observed-minus-calculated residuals relative to the predicted pass (corrected by the time bias applied to center the target) are displayed in Fig. 1. The longest measurement lasted for approximately 100 s. A slope within the reflected photons indicates that the time bias used to center the target was slightly underestimated, which is related to imperfect alignment of the optical axis of the piggyback telescope. Due to the remaining time bias, the object moves to regions further away from the initial detector activation time and the trace of the debris within the noise would soon disappear. Once recognizing the returns from the object the observer shifts the triggering time towards the returning photons (to longer times), increasing the detection probability (Fig. 1b–d). If the observer shifts the activation time in the wrong direction, the rocket body is lost within the noise (Fig. 1a). A correct time bias results in returns appearing as a straight line within the residuals (Fig. 1d). To verify that the data is coming from the whole rocket body a close-up of two passes (c, d) is shown (Fig. 1e, f). Applying the time and range biases to the predicted orbit, it is possible to match the measured values to predicted ones resulting in residuals close to 0. This allows to see fine structures and estimate a minimum size of objects. Returns coming from the front and the back of the body were detected corresponding to range differences of up to 8 m giving an indication of the size of the targets.

## Discussion

Due to the large offsets of space debris targets with respect to the available predictions it is necessary to visualize them with optical telescopes and cameras. During night time Low Earth Orbit satellites are only visible within a few hours around sunset or sunrise because the satellite needs to be illuminated by the sun while not being shadowed by Earth. This restriction in observation time is partially overcome by making large space debris objects visible during daylight. A suitable combination of telescope, detector and filter was chosen to increase the contrast of objects with respect to the daylight sky. Stars up to a magnitude of 8 and more than 40 different space debris objects were observed. The path of a tracked Earth orbiting object in the field of view of a tracking camera was analyzed with respect to along-track, across-track and range offsets. A software was developed which detects illuminated objects and calculates the offset of targets with respect to the predictions. These calculations were then used to center the target within the field of view of the SLR receiving telescope before the SLR search routine was started. Space debris laser ranging echos were presented for four different upper stage rocket bodies.

**Fig. 2:**
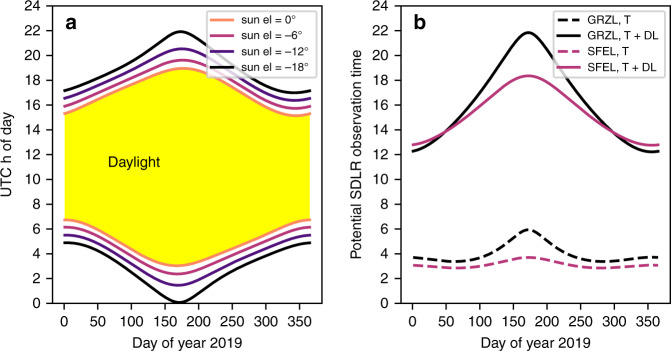
**Twilight phases and potential space debris observation time**. **a** Astronomical, nautical, and civil twilight for Graz during the year 2019. The twilight phases correspond to sun elevations of  −18°,  −12°, −6°, and 0°. **b** The total potential time of space debris laser ranging observation time increases in Graz (GRZL, black, latitude 47.1°N) to up to 22 h including the twilight (T) phase and full daylight (DL). At the lower latitude San Fernando laser ranging station (SFEL, red, latitude 36.5°N) potential observation times increase to approximately 18 h.

These daylight space debris laser ranging results guide the way to significantly increasing potential observation times. Depending on the season, for the Graz SLR station twilight conditions occur for a maximum of 6 h per day while daylight lasts for 8–16 h (Fig. 2a), increasing potential observation times in Graz to up to 22 h (Fig. 2b). Due to the lower duration of the twilight phases potential observation times for lower latitude SLR stations (e.g. San Fernando at 36°N) are slightly reduced. This clearly points out that including twilight and full daylight will drastically increase the output of all stations capable of doing space debris measurements. The increased coverage will encourage an observation network of space debris stations^25^ to be formed (similar to the International Laser Ranging Service^26^) which could immediately react in case of conjunction warnings targeting a certain object rapidly improving the predictions. Improved predictions are central in decision-making with respect to avoidance maneuvers. In addition to that, highly accurate orbits are crucial for future active removal^27^ or laser nudging^28^ missions.

## Methods

### A guideline to daylight observations

The visibility of space debris objects during daytime is limited by the contrast against the sky background. The contrast and brightness on a detector is influenced by the diffraction limit of the optics, objects’ dimensions, the field of view per detector pixel, atmospheric seeing^29^, and the exposure time of the detector. To reach optimal contrast the telescope aperture should be chosen large enough to minimize the size of the Airy disk on the sensor. The angle of the first Airy minimum (*Θ*_*a*_) of a star is connected to the wavelength of light *λ* and the aperture diameter of the telescope *d* via *Θ*_*a*_ = 1.22*λ*/*d*.

Reducing the size of the Airy disk (increasing the telescope aperture) while staying in perfect focus maximizes the light concentrated on each individual pixel, directly increasing contrast at a given detector field of view. The detector and focal length of the telescope should be chosen in a way that the field of view per pixel matches the angular size of the object on the sky. A field of view per pixel larger than the object size on the sensor (undersampling) will decrease the contrast as the pixel relatively gathers more skylight than light from the object. Reducing the field of view per pixel (*f*_*p*_) below the object size will equally decrease skylight and object light and the contrast will remain unaffected. Due to atmospheric turbulences at very short exposure times in the order of a few milliseconds the image shows speckles. These individual distorted images of the star correspond to the current atmospheric conditions. Speckles also distribute the intensity of the object across the sensor but each individual speckle will remain diffraction limited. The field of view per pixel should hence be fitted to the Airy disk and not to the seeing. What should be avoided though are longer exposure times which let pixels accumulate sky light while not being exposed to starlight.

In conclusion, for optimal contrast increasing the aperture of the telescope is beneficial in reducing the Airy disk of the object. The detector field of view per pixel should be chosen to match the Airy disk. Seeing will reduce the object brightness but will have low influence on contrast as long as the exposure time is short enough to freeze the current atmosphere.

**Fig. 3:**
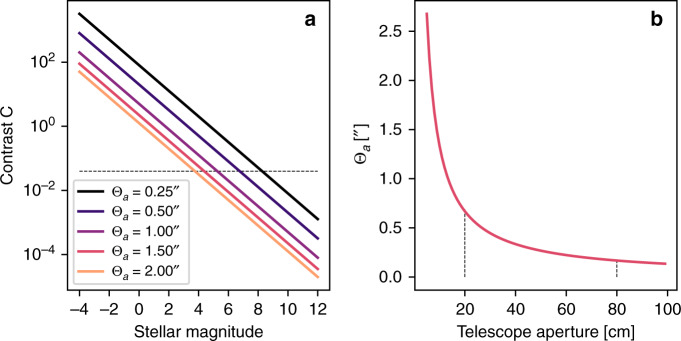
**Sky contrast vs. stellar magnitude, Airy minimum vs. telescope aperture**. **a** Contrast C of stars with different magnitudes against a sky background of ${{m}}_{{\rm{sky}}}=3\ {\rm{mag}}\ {{\rm{arc}}\,{\rm{s}}}^{-2}$. The dashed line corresponds to an estimated contrast detection limit of 0.04. **b** Angle of the first Airy minimum (*Θ*_*a*_) vs. telescope aperture. The dashed lines highlight the Airy limit for the used telescopes with 20 and 80 cm aperture.

Assuming a star whose brightness is uniformly distributed within the first Airy minimum (*Θ*_*a*_, Fig. 3) and scaling the magnitude of the sky (*m*_sky_) and star (*m*_star_) down to one pixel while applying the logarithmic relation between magnitude and flux density (*F*) leads to a simple formula for the contrast ratio *C* between stellar (*F*_star_) and sky flux density (*F*_sky_) during daylight observations.

1 $$C=\frac{{{F}}_{{\rm{star}}}}{{{F}}_{{\rm{sky}}}}\approx \frac{1}{{{A}}_{{\rm{star}}}}1{0}^{\frac{{{m}}_{{\rm{sky}}}-{{m}}_{{\rm{star}}}}{2.5}}\approx \frac{1}{{\Theta }_{{a}}^{2}\pi }1{0}^{\frac{{{m}}_{{\rm{sky}}}-{{m}}_{{\rm{star}}}}{2.5}}.$$

The units of the Airy minimum and the sky brightness are given in arc s and mag arc s^−2^, respectively. The contrast between star and sky is hence reduced by a factor dependent on the area of the star in the image plane of the telescope (*A*_star_) and hence on the Airy disk. The contrast was evaluated for an estimated sky brightness of ${{m}}_{{\rm{sky}}}=3\ {\rm{mag}}\ {{\rm{arc}}\,{\rm{s}}}^{-2}$ and different Airy limits (*Θ*_*a*_). The dashed line corresponds to an estimated contrast detection limit of 0.04. This equals a pixel intensity difference of 5 at a background intensity of 125, within a range of 0–255.

### Visualization of stars and rocket bodies

Two different sensors were available for daylight observations of stars and satellites: ZWO ASI 120 (4.8 mm × 3.6 mm, 3.8 μm pixels, 1280 × 960 pixels), ZWO ASI 1600 (17.7 mm × 13.4 mm, 3.8 μm pixels, 4656 × 3520 pixels). These were attached to an 80 cm Ritchey–Chretien telescope with a focal length of *f* = 4.8 m (*Θ*_*a*_ = 0.17*″*, *f*_*p*_ = 0.16*″*). For visualizing satellites during SLR tracking a 20 cm Schmidt–Cassegrain telescope (*Θ*_*a*_ = 0.69*″*, *f*_*p*_ = 0.39*″*) was piggyback-mounted on our 50 cm SLR receive telescope. A 780 nm long pass filter was used to reduce daytime skylight. A real-time image analysis software was developed to automatically detect space debris during daylight and—based on the predicted path—estimate time biases (the temporal offset along satellite track) and across-track errors. During SLR tracking the biases are used to correct tracking (the predicted path) and to adapt the telescope pointing. During daylight conditions with increased noise and low accuracy of the predictions, a fast response of the whole laser ranging system is essential.

**Fig. 4:**
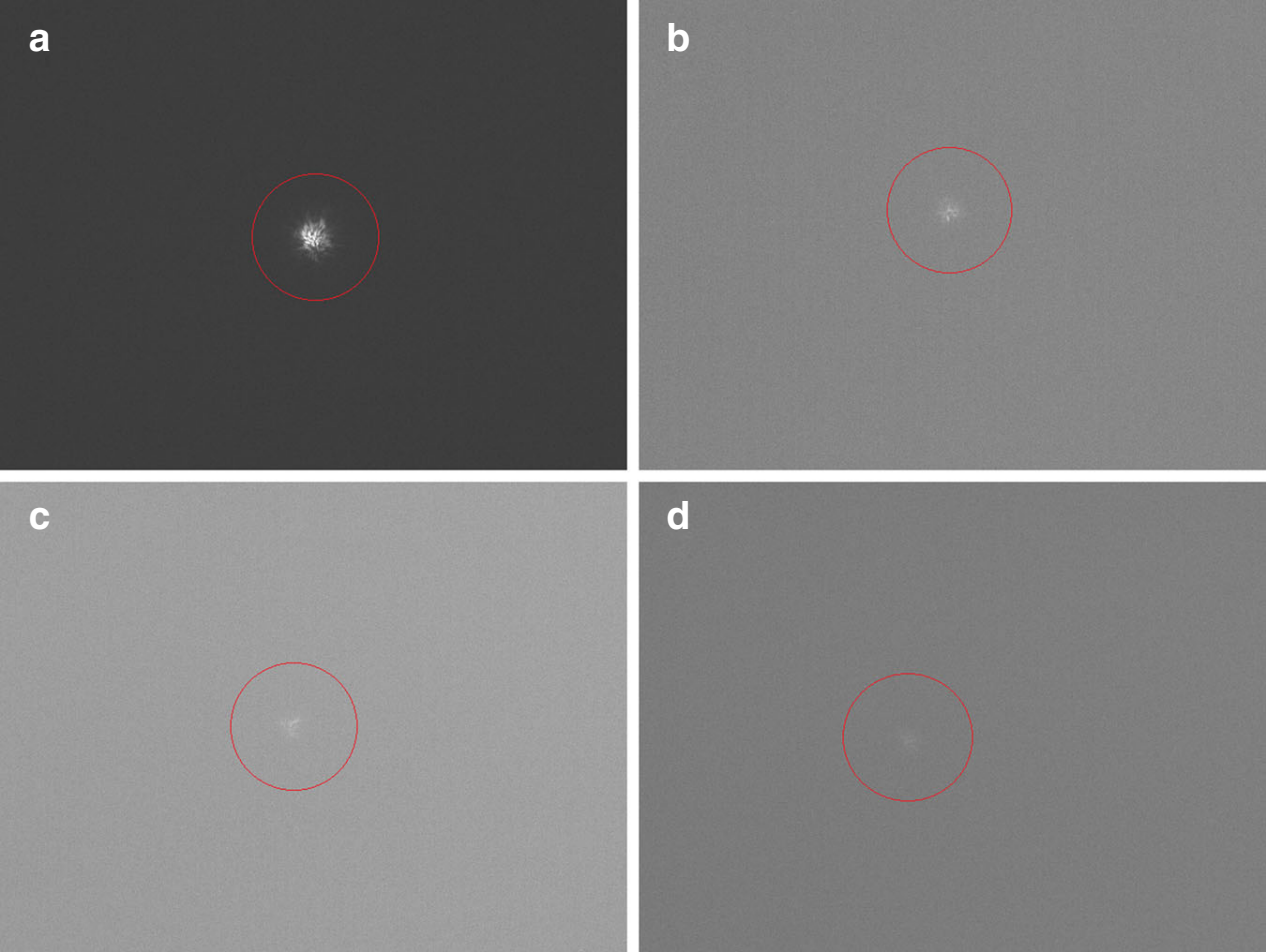
**Visualization of stars during daylight**. Daylight observations of stars of the Hipparcos (HIP) catalog with magnitudes (m). The images were taken with an ASI120 between 12:14 and 13:43 UTC at sun elevations between 10° and 18°. The images were cropped to a field of view of 1.7 × 1.1 arc min. Red circles highlight the star images for easier recognizability. **a** HIP69673 (*m* = 0.15, *az* = 255°, *el* = 39°), **b** HIP71075 (*m* = 3.00, *az* = 277°, *el* = 50°), **c** HIP81646 (*m* = 6.95, *az* = 340°, *el* = 60°), **d** HIP91915 (*m* = 8.25, *az* = 345°, *el* = 75°).

The limiting conditions for star observations during daylight were tested by pointing the telescope to different stars with decreasing brightness. Stars with magnitudes between 0.15 and 8.25 were captured with an ASI 120 on 2018/11/28 at sun elevations ranging from 10° to 18° (Fig. 4). All of them were also detected by the real-time software. Comparing the results to the theory (Fig. 3) it can be seen that the magnitude limit corresponds well with the predictions (*Θ*_*a*_ = 0.17*″* for *d* = 80 cm).

**Fig. 5:**
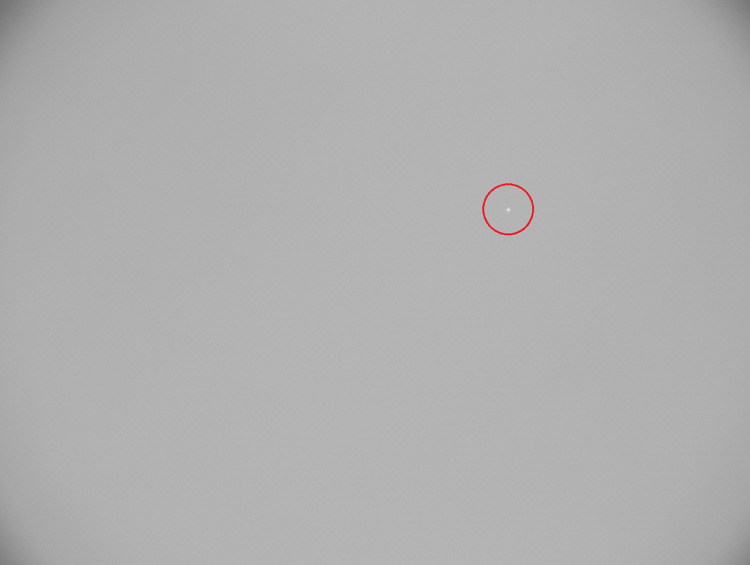
**Visualization of a rocket body during daylight**. Sunlight reflections of an SL-12 rocket body (NORAD ID: 15772) displaying a field of view of 0.21° × 0.16°. The image was captured with an ASI 1600 on 2018/12/04, 14:31 UTC at a sun elevation of 5°. A red circle highlights the rocket body for easier recognizability.

For the visualization of rocket bodies the larger ASI 1600 camera was used to account for offsets due to inaccurate TLE predictions. The sunlight reflections of an SL-12 rocket body (NORAD: 15772) are easily visible on the captured image (Fig. 5a). The importance of sensors corresponding to a large field of view becomes clear immediately. Due to the large offsets of the TLE predictions with respect to the true orbit the object appears in the outer regions of the sensor. On the smaller ASI 120 sensor it would have been outside the field of view. Overall, more than 40 different upper stage rocket bodies were visualized with this technique during daylight.

### Space debris laser ranging equipment

For all space debris laser ranging measurements the following equipment was used: the space debris laser operates at 532 nm firing 80 mJ laser pulses with 3 ns pulse length at 200 Hz with 16 W. To minimize the beam divergence, the laser beam is expanded to 7 cm diameter. The laser beam divergence ($2\ {\rm{arc}}\,{\rm{s}}$, half angle) corresponds well to the typical astronomical seeing at our station. A 50 cm diameter receive telescope is used to detect the reflected photons and focuses the incoming light to a C-SPAD: Compensated Single Photon Avalanche Diode detector^30^ with 200 μm diameter. It is operated in a gated mode; the detector is activated with respect to the predicted arrival time of the reflected photons. The activation pulses are set via a field-programmable gate array evaluating the laser start pulse to generate predicted arrival times (range gate generator).

After activation of the SPAD the detector will be triggered due to dark noise or sky noise within a few microseconds. During daylight laser ranging the limiting factor will not be intrinsic detector noise but the sky background which follows Poisson statistics. The probability of the detection of a reflected photon within the noise is hence increased the sooner the return photon arrives after SPAD activation. To decrease noise during daylight laser ranging the field of view of the receive telescope is limited to approximately 100 μrad and filters are used to limit the spectral bandwidth.

Graz’ space debris target catalog includes more than 200 different objects, mostly upper stage rocket bodies and nonfunctional satellites. The radar cross-section ranges from 0.3 to 15 m^2^, the orbital heights begin from a few hundred up to approximately 1500 km.

### Reporting summary

Further information on experimental design is available in the Nature Research Reporting Summary linked to this paper.

## Supplementary information

Supplementary Information

## Data Availability

The datasets generated during and/or analyzed during the current study are available from the corresponding author on reasonable request.

## Code availability

The code for the post processing of the datasets written in Python is available on reasonable request. The software written for the automatic detection and time bias calculation of targets written in Labview^31^ is available on reasonable request.
