# Supplementary material for: Daylight space debris laser ranging
Source: Nat Commun. 2020 Aug 4;11:3735. doi: 10.1038/s41467-020-17332-z (PMC7403592; doi:10.1038/s41467-020-17332-z)
Supplement: Supplementary file 1 — Supplementary Information [file 41467_2020_17332_MOESM1_ESM.pdf]

REVIEWERS' COMMENTS:

Reviewer #1 (Remarks to the Author):

As you know, single-shot precision of space debris laser ranging can achieve up to the level of decimeter for space debris, higher 1 to 2 orders of magnitude than microwave radar and optical tracking technologies. It is very helpful for precise orbit determination, orbit check and cataloging of space object and will also be important for enhancing the capabilities of collision avoidance warning and building a dynamic database for tracked space debris. In addition, compared with the optical tracking technologies, laser ranging technology can be used to observe space debris in earth shadow or daytime due to monochromatic, which will be helpful for extending the observation arc.

More than 95% of space debris were in low earth orbit (LEO), their orbital periods are about one and half an hour, it passed the observation station quickly and the passed time is about a few minutes. Space debris are only visible within a few hours around sunset or sunrise. In this manuscript, stars with magnitude of 8 and more than 40 different space debris objects were visualized during daylight by using 780nm long pass filter and the field of view per pixel equaling to the angular size of the object. Inaccurate predictions of about 1km was improved by a real time detection software and 532nm firing 80mJ laser pulses with 3ns pulse length at 200Hz with 16W was used and the laser beam is expanded to 7cm diameter. A 50 cm diameter receive telescope is used to detect the reflected photons and focuses the incoming light to a C-SPAD. To decrease noise during daylight laser ranging the field of view of the receive telescope is limited to approx. 100μrad and filters are used to limit the spectral bandwidth. Therebefore, several rocket bodies of space debris were measured during daylight.

It’s very useful to space debris for removal missions, conjunction warnings and avoidance maneuvers.

The job of this article is an innovative and great work for space debris laser ranging because it is the first space debris laser ranging results during daylight.

I suggest this manuscript published.

Reviewer #2 (Remarks to the Author):

This is a thorough piece of work that takes forward the well-established geodetic technique of satellite laser ranging (SLR) into the very active field of space debris tracking. In particular the work builds upon the existing knowledge that laser ranging, using sufficiently powered lasers, can be applied to 'non-cooperative' targets that do not carry retro reflectors and demonstrates clearly that the technique can be used during the daytime. This is technically challenging work, where the authors have solved poor signal-to-noise issues in order to detect optically the target satellites as well as in realtime correcting the omnipresent errors in existing orbital predictions; both these steps are required in order to obtain sufficient laser returns that unambiguous range measurements may be secured and extracted from the noise background that is inevitable in daytime conditions. The authors point out that this advance to tracking debris in daytime means that a given laser station will vastly increase its yield of observations of this important class of orbiting object. The detail of the techniques employed are clearly set out, such that other interested tracking stations might rapidly get up to speed; the authors offer their data and algorithms to the interested members of the space debris community, which is to be applauded. Just a few typos and suggestions for better wording have been marked on the annotated manuscript. Graham Appleby

Reviewer #3 (Remarks to the Author):

This is an excellent paper, which should be published. It is good solid scientific work and represents well what can be done with laser ranging starting from an Earth based station. My only regret is that the authors did not at least mention the option of a space based laser station which will have none of the disadvantages of atmospheric distortion, limited observation time, atmospheric nonlinear refraction, larger background signal, etc. I appreciated the authors mentioning the goal of matching projected pixel area to the size of the target. I especially enjoyed their discussion of all the detectors and lasers now available for this work. Their data is excellent.

Response to the referees:

Reviewer #1 (Remarks to the Author):

As you know, single-shot precision of space debris laser ranging can achieve up to the level of decimeter for space debris, higher 1 to 2 orders of magnitude than microwave radar and optical tracking technologies. It is very helpful for precise orbit determination, orbit check and cataloging of space object and will also be important for enhancing the capabilities of collision avoidance warning and building a dynamic database for tracked space debris. In addition, compared with the optical tracking technologies, laser ranging technology can be used to observe space debris in earth shadow or daytime due to monochromatic, which will be helpful for extending the observation arc.

More than 95% of space debris were in low earth orbit (LEO), their orbital periods are about one and half an hour, it passed the observation station quickly and the passed time is about a few minutes. Space debris are only visible within a few hours around sunset or sunrise. In this manuscript, stars with magnitude of 8 and more than 40 different space debris objects were visualized during daylight by using 780nm long pass filter and the field of view per pixel equaling to the angular size of the object. Inaccurate predictions of about 1km was improved by a real time detection software and 532nm firing 80mJ laser pulses with 3ns pulse length at 200Hz with 16W was used and the laser beam is expanded to 7cm diameter. A 50 cm diameter receive telescope is used to detect the reflected photons and focuses the incoming light to a C-SPAD. To decrease noise during daylight laser ranging the field of view of the receive telescope is limited to approx. 100µrad and filters are used to limit the spectral bandwidth. Therebefore, several rocket bodies of space debris were measured during daylight.
It’s very useful to space debris for removal missions, conjunction warnings and avoidance maneuvers.
The job of this article is an innovative and great work for space debris laser ranging because it is the first space debris laser ranging results during daylight.
I suggest this manuscript published.

Answer: We thank the reviewer for the positive answer and are happy to share the results with the community.

Reviewer #2 (Remarks to the Author):

This is a thorough piece of work that takes forward the well-established geodetic technique of satellite laser ranging (SLR) into the very active field of space debris tracking. In particular the work builds upon the existing knowledge that laser ranging, using sufficiently powered lasers, can be applied to 'non-cooperative' targets that do not carry retro reflectors and demonstrates clearly that the technique can be used during the daytime. This is technically challenging work, where the authors have solved poor signal-to-noise issues in order to detect optically the target satellites as well as in realtime correcting the omnipresent errors in existing orbital predictions; both these steps are required in order to obtain sufficient laser returns that unambiguous range measurements may be secured and extracted from the noise background that is inevitable in daytime conditions. The authors point out that this advance to tracking debris in daytime means that a given laser station

will vastly increase its yield of observations of this important class of orbiting object. The detail of the techniques employed are clearly set out, such that other interested tracking stations might rapidly get up to speed; the authors offer their data and algorithms to the interested members of the space debris community, which is to be applauded. Just a few typos and suggestions for better wording have been marked on the annotated manuscript. Graham Appleby

Answer: We thank the reviewer for the positive answer and implemented the comments in our revised manuscript. Below, the changes to the document are listed point-by-point. The line numbers correspond to the document “2_reviewer_attachment_1_1585502074.pdf” and the new document (in brackets)

Line 57 (Line 48): The apostrophe was added to the word objects’

Line 76 (Line 67): The sentence was changed to “increasing the aperture of the telescope is beneficial in reducing the Airy disk of the object”

Line 107 (Line 98): typo: “in” was changed to “it”

Line 107 (Line 98): naming: “r_a” was changed into “theta_a”

Line 138 (Line 130): wording: “8-inch” was changed to “20cm”

Line 142 (Line 134): wording: “favorably” was changed into “primarily”

Line 142 (Line 135): wording: “due to TLE errors” was added to the sentence

Line 143 (Line 136): wording: ”to the predictions” was added to the sentence

Line 146-147 (Line 139-140): wording: “experimentally” was added to the sentence, “accordingly” was removed

Line 166 (Line 159): typo: “minimal” was changed to “minimum”

Line 174 (Line 166): wording: changed to “This restriction in observation time is partially overcome…”

Line 184 (Line 177): wording: The sentence was changed to: “Depending on the season, for the Graz SLR station…”

Line 188 (Line 181): wording: “36°N” was added to the sentence. Furthermore in the caption of Figure 5 “N” was added as well

Line 193 (Line 186): wording: changed to “decision-making”

Line 193 (Line 186): wording: a comma was added to the sentence: “In addition to that, highly accurate orbits are crucial…”

Reviewer #3 (Remarks to the Author):

This is an excellent paper, which should be published. It is good solid scientific work and represents well what can be done with laser ranging starting from an Earth based station. My only regret is that the authors did not at least mention the option of a space based laser station which will have none of the disadvantages of atmospheric distortion, limited observation time, atmospheric nonlinear

refraction, larger background signal, etc. I appreciated the authors mentioning the goal of matching projected pixel area to the size of the target. I especially enjoyed their discussion of all the detectors and lasers now available for this work. Their data is excellent.

Answer: We would like to thank the reviewer for the constructive comment.

A space-bound laser ranging station indeed is a very interesting technique with the most prominent advantage being the lack of atmospheric distortion. We have had discussions within our community about such a station concept. As to our knowledge no station is currently developing/planning such a station.

However, the most important disadvantage is the cost of such a station. In addition to the expensive rocket launch, maintenance will be complicated and components have to be space qualified. It might however be possible to reduce overall size of the system due to the lack of noise. Although there is some tendency towards automation of SLR stations within the community, it is still necessary to perform regular maintenance work and adjustments on-site.

Nowadays the trend within the laser ranging community goes into the direction of a modular station, built out of "commercial of the shelf" components. In our opinion the future of satellite laser ranging will be to have a larger network of high-quality ground based stations, which are easier to maintain. A good example of such a station is currently built on Tenerife, which allowed to reduce the costs drastically as compared to Coudé path based stations.

Overall we see space-based laser ranging as a very interesting but also very challenging task, which might give new insights to the community in the near future.
